# Examination of the Virulence of *Actinobacillus pleuropneumoniae* Serovar 16 in Pigs

**DOI:** 10.3390/vetsci11020062

**Published:** 2024-02-01

**Authors:** Miklós Tenk, Gergely Tóth, Zsuzsanna Márton, Rita Sárközi, Alejandra Szórádi, László Makrai, Nimród Pálmai, Tamás Szalai, Mihály Albert, László Fodor

**Affiliations:** 1 CEVA-Phylaxia Veterinary Biologicals Co., Ltd., Szállás u. 5, H-1107 Budapest, Hungary; tenk.miklos@univet.hu (M.T.); zsuzsanna.marton@ceva.com (Z.M.); maria-alejandra.szoradi@ceva.com (A.S.); nimrod.palmai@ceva.com (N.P.); tamas.szalai@ceva.com (T.S.); mihaly.albert@ceva.com (M.A.); 2 Department of Microbiology and Infectious Diseases, University of Veterinary Medicine, Hungária Krt. 23-25, H-1143 Budapest, Hungary; toth.gergely@univet.hu (G.T.); rita.sarkozi@gmail.com (R.S.); autovakcina@gmail.com (L.M.)

**Keywords:** *Actinobacillus pleuropneumoniae*, serovar 16, challenge

## Abstract

**Simple Summary:**

*Actinobacillus (A.) pleuropneumoniae* is a major agent of the porcine respiratory diseases complex (PRDC), together with several bacterium and virus species. *A. pleuropneumoniae* strains are diverse regarding virulence and antigen structure; they have different virulence variants, and the strains can be assigned into 19 serovars. Serovar 16 was described in Hungary for the first time, and it is relatively frequent in pig herds. The aim of the present work was examination of the virulence of the *A. pleuropneumoniae* A-85/14 strain, the type strain of serovar 16, by infecting piglets. The results prove that the *A. pleuropneumoniae* A-85/14 strain is a virulent one.

**Abstract:**

Different virulence variants of *A. pleuropneumoniae* are involved in the etiology of porcine pleuropneumonia. The purpose of the present trial was examination of the virulence of the *Actinobacillus pleuropneumoniae* A-85/14 strain, the type strain of serovar 16, in an animal challenge experiment. Thirty 12-week-old piglets seronegative for *A. pleuropneumoniae* were allocated into three trial groups each of 10 animals, and they were infected intranasally with 10^6^, 10^7^, or 10^8^ colony forming units (cfu) of the strain, respectively. Clinical signs were recorded twice a day, and the animals were euthanized 6 days after the infection. Typical clinical signs and postmortem lesions of porcine pleuropneumonia were seen in the animals of each trial group; however, they were generally mild, and no significant differences could be seen between the three groups. Even 10^6^ colony forming units of *A. pleuropneumoniae* A-85/14 strain could induce clinical signs and lesions. Based on these results, the type strain of serovar 16 of *A. pleuropneumoniae* must be regarded as a typical pathogenic strain of the species.

## 1. Introduction

Porcine respiratory disease complex (PRDC) is a major health concern of the swine industry all over the world. The pathogenicity of the disease is complex; several agents, like porcine circovirus 2 (PCV-2), porcine reproductive and respiratory syndrome virus (PRRSV), Aujeszky disease virus (*Suid herpesvirus 1*), swine influenza virus, *Mycoplasma hyopneumoniae*, *Mycoplasma hyorhinis*, *Pasteurella multocida*, *Glaesserella parasuis*, and *Actinobacillus (A.) pleuropneumoniae*, are involved in the etiology, however, different environmental factors, nutrition, and management problems can predispose the animals to the disease or can trigger the clinical signs. PRDC can be the reason for a high morbidity and mortality of pigs, and decreased profitability of piggeries [1,2,3,4]. The etiological impact of the above agents can be different in the certain farms, but *A. pleuropneumoniae* strains frequently have a dominant role.

*A. pleuropneumoniae* was first isolated in Great Britain [5], and its pathogenic importance was confirmed worldwide; it is widely distributed in the swine population [2], including Hungary [6,7,8]. Different serovars of *A. pleuropneumoniae* are carried in the tonsils and upper respiratory tract of pigs, and as a result of external and internal predisposing factors and stress, they enter into the lower respiratory tract and cause fibrino-hemorrhagic, necrotizing pneumonia, pulmonary oedema, and fibrinous pleuritis disease, sometimes with multifocal abscesses in growing and fattening pigs. In some animals, a chronic form of the disease can be seen, characterized by retarded growth and localized lesions in the lungs [2,9]. *A. pleuropneumoniae* can also be carried by wild boars, however, they do not seem to be reservoir animals [10]. *A. pleuropneumoniae* is regarded as adapted to the respiratory tract, and lesions caused by *A. pleuropneumoniae* are generally limited to the lungs, but the agent can sometimes cause generalized diseases [2,11,12]. The etiologic role of *A. pleuropneumoniae* strains was sporadically confirmed in the case of meningitis, nephritis [13], multifocal granulomatous hepatitis [14], and necrotizing osteomyelitis and fibrinopurulent arthritis [15].

Porcine actinobacillosis has a great economic impact due to losses, costs of medication, increased fattening period, decreased body mass gain, and food conversion [16]. Different antibiotics are used to decrease the economic losses of the disease [17,18].

*A. pleuropneumoniae* has two biotypes; biotype I strains need nicotinamide adenine dinucleotide (NAD, V-factor) to grow, while biotype II strains are not dependent on NAD. Several virulence factors of *A. pleuropneumoniae* have been identified; a combination of four types of pore-forming Apx toxins (RTX toxins) are regarded as major ones. The ApxIV toxin is produced by all isolates of *A. pleuropneumoniae* but not by other species of the *Actinobacillus* genus [19]. The ApxI toxin is strongly hemolytic and cytotoxic for alveolar macrophages and neutrophils; the ApxIII toxin is non hemolytic and has a slightly lower toxic activity, while the weakly hemolytic ApxII toxin that is produced by the majority of the serovars has moderate toxic activity but synergistically increases the effect of the other Apx toxins [20]. Fimbria, outer membrane proteins, lipopolysaccharides, polysaccharides, ability of biofilm formation, presence of transporter systems, and different enzymes can also facilitate *A. pleuropneumoniae* in causing disease. The variety of the virulence factors results in great differences in the virulence of the strains [21,22,23,24,25,26,27].

The only host species of *A. pleuropneumoniae* is the swine, and hence the virulence properties of an *A. pleuropneumoniae* strain can only be measured in animal trials by infecting this target species. Several infection models have been used. Pigs were infected with *A. pleuropneumoniae* by intratracheal [9,28] intranasal [29,30,31], intradermal or subcutaneous [32], and aerosol [33,34] ways. Mouse infection assays were also described using intranasal [35] and intraperitoneal challenge [36]. A simple infection model using *Galleria mellonella* wax moth was applied by Pereira et al. (2015), however, it did not become widely used [37].

A total of 19 serovars have been identified so far, on the basis of surface-soluble capsular polysaccharide antigens [38]. The geographical distribution of different biotypes and serovars of *A. pleuropneumoniae* shows a distinct pattern. Serovar 2 is dominant in most European countries and Japan [8,39,40,41,42]. Serovar 5 and 7 were the most frequent ones in Canada [43]. Serovar 16 was described in Hungary, and it was isolated in several swine herds in the country from animals showing typical clinical signs and lesions of Actinobacillus pleuropneumonia; furthermore, 8.8% of the isolated *A. pleuropneumoniae* strains were allocated into this serovar [8,44]. Serovar 16 isolates formed a single cluster when examined with pulsed-field gel electrophoresis [45].

After characterizing the antigenic and genetic properties of *A. pleuropneumoniae* strain A-85/14 [44,46], the type strain of serovar 16, the aim of the present work was examination of the virulence of this strain, by intranasally infecting piglets with three dilutions of it.

## 2. Materials and Methods

### 2.1. Animals

Thirty 12-week-old male and female meat-type Hungarian Large White piglets from a controlled breeding environment, seronegative for *A. pleuropneumoniae,* were enrolled in the trial. They were housed in isolated rooms of the minimal disease animal house of the Department of Microbiology and Infectious Diseases on deep litter, without contact to other pigs. They received commercial feed and ad libitum tap water. The body mass of the piglets was measured the day before the infection and before euthanasia of the animals. Randomization was based on body mass, and three groups (Groups 1–3) with 10 animals each were formed.

### 2.2. Experimental Design

The animals were infected after a week-long acclimatization. *A. pleuropneumoniae* strain A-85/14 was propagated on chocolate agar made of tryptone soya agar (TSA, Biolab Ltd., Budapest, Hungary) with added 50 μg/mL NAD (Biolab Ltd., Budapest, Hungary) (TSA-NAD) at 37 °C for 18 h. The challenge solutions were prepared using the inoculum from this 18 h agar plate. A 250 mL shake flask with tryptic soy broth (Merck, Rahway, NJ, USA) supplemented with 50 μg/mL NAD (Sigma, St. Louis, MO, USA) (TSB-NAD) was inoculated with fresh culture. The broth was then incubated in a shaker thermostat at 37 °C with continuous agitation at 100 RPM (rounds per minute) to provide a sufficient oxygen saturation. The incubation was stopped when the colony forming unit (cfu) count reached 10^8^ cfu/mL, based on the preceding photometric cfu calibration study. Then, with 10-fold dilutions in TSB-NAD, three suspensions were prepared, containing 10^7^, 10^6^, and 10^5^ cfu/mL. The cell count was checked before and after the challenge by plating onto TSA-NAD. All trial animals received 5 mL of bacterium suspension into each nostril (Group 1: 10^8^ cfu/animal; Group 2: 10^7^ cfu/animal; Group 3: 10^6^ cfu/animal). Piglets of the same age on the farm of origin served as controls. Six days after the infection, the animals were euthanized using electric stunning and exsanguination. Clinical signs, body mass gain, and postmortem lesions were evaluated. The study was conducted in compliance with the provisions of Directive 2010/63/EU, Hungarian Act XXVIII/1998, the Hungarian Ministerial Decree No. 40/2013. (II. 14.) and the permission (PE/EA/3340-6/2016) issued by the Governmental Office of Pest County, Hungary.

### 2.3. Clinical and Postmortem Examinations

Clinical signs were observed and recorded two times a day, for 7 days. They were scored as presented in Table 1.

Cumulative clinical scores, length of clinical signs, number of febrile days, and body mass gain were evaluated, following the methods used in vaccine efficacy trials [47]. All animals were subjected to necropsy, and organs were sampled by histology and bacteriological examination. Each lung lobe was scored (0–5) according to the percentage of consolidated lung masses: 1 score (1–20%), 2 (21–40%), 3 (41–60%), 4 (61–80%), 5 (81–100%) [48]. Lung samples obtained at necropsy were fixed in 4% neutral buffered formalin for 48 h, embedded in paraffin wax, sectioned at 4 μm, and stained with hematoxylin and eosin (HE) [49].

### 2.4. Serological and Bacteriological Examinations

Serum samples of all animals collected before challenge were tested with ELISA (APP-ApxIV Ab Test, IDEXX) according to the instruction of the producer.

Samples from lungs and mediastinal lymph nodes were inoculated on blood agar plates nursed with *Staphylococcus aureus* and the plates were incubated at 37 °C for 24 h. The isolated strains were identified on the basis of their cultural, morphological, and biochemical characteristics [50]. The identification was confirmed with a PCR test detecting the gene of the ApxIV toxin [10]. They were serotyped in a passive hemagglutination test using hyperimmune sera raised in rabbits against 1–19 serovar type strains of *A. pleuropneumoniae* as described in [8,44].

### 2.5. Statistical Evaluation

The data of the body mass, clinical, and postmortem scores (average value, standard deviation, Student’s *t*-test) were compared using the Unscrambler 10.3 program (CAMO Software AS., Oslo, Norway). The statistical unit was the individual animal.

## 3. Results

### 3.1. Clinical Signs

The first clinical signs appeared in all three groups 6 h after the infection; however, they were not very severe. Some animals were depressed, reluctant to move, and lay in one group. Decreased appetite, cough, nasal discharge, and dyspnea were seen, and this lasted for 3–5 days in the case of Group 1 and 2 pigs, while pigs in Group 3 showed clinical signs for 1–2 days. The average cumulative clinical scores, length of clinical signs, and number of febrile days are presented in Table 2. Only one animal died during the trial. One piglet from Group 3 died a few hours after the infection, and shock was diagnosed in the postmortem examination, so it was not included in the trial. Control animals in the original herd remained healthy during the observation period.

### 3.2. Body Mass and Mass Gain

The body mass and the mass gain are presented in Table 3. There were considerable individual differences within the trial groups; however, there were no significant differences between the groups at the beginning of the trial. At the end of the observation, the lowest average body mass and mass gain were seen in Group 1, which was infected with the highest number of bacteria, while both body mass and mass gain were higher in the groups that received less bacteria. The differences were not significant with the exception of body mass between Group 1 and 3 at the end of the trial.

### 3.3. Postmortem Lesions

**Figure 1:**
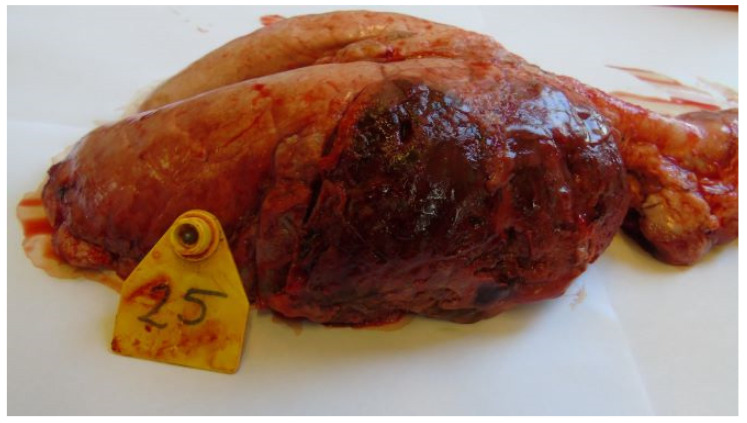
Extended hemorrhagic, necrotic pneumonia (Group 3).

**Figure 2:**
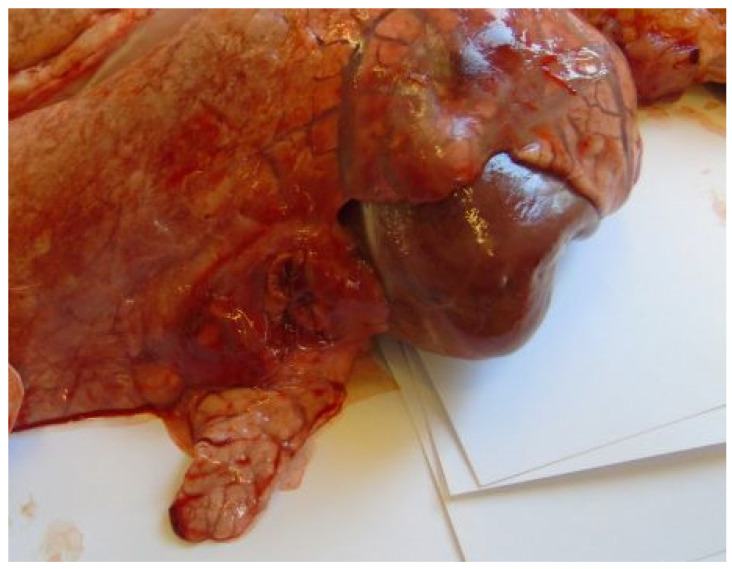
Focal necrotic pneumonia (Group 2).

Most animals had postmortem lung lesions. The extent of the lung lesions was variable; severe and mild lesions were seen in all groups, and there were no significant differences in the postmortem scores between the groups. In most cases, acute fibrino-hemorrhagic, necrotic pneumonia, fibrinous pleurisy, and oedema could be observed. Both extended lesions (Figure 1) and focal ones with sequester (Figure 2) could be seen. *A. pleuropneumoniae* serovar 16 was re-isolated in a large number from most lung lesions and mediastinal lymph nodes (Table 4). The row data are presented in the in the Supplementary Table S1.

### 3.4. Histopathology

**Figure 3:**
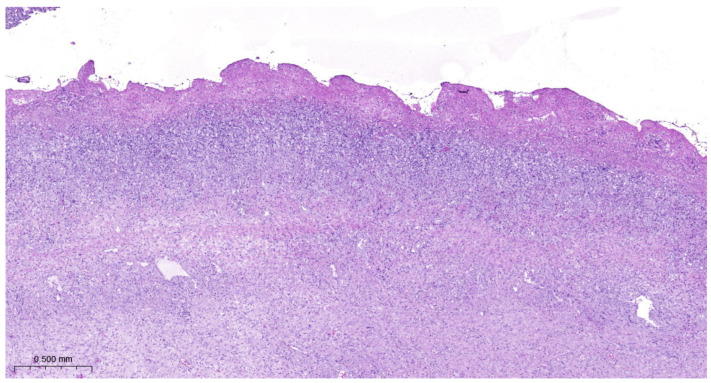
*A. pleuropneumoniae* serovar 16-infected piglet’s pleura on 6th day postinfection. Organization of fibrin. Granulation tissue on the pleural surface. H.E. staining.

**Figure 4:**
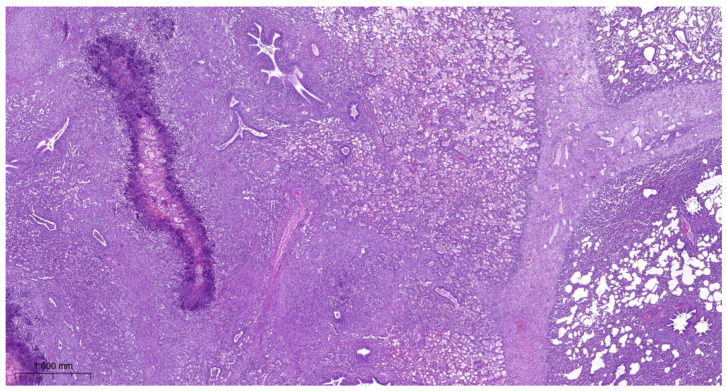
*A. pleuropneumoniae* serovar 16-infected lung on 6th day postinfection. Focal necrotic area in the lung surrounded by mononuclear inflammatory cells. Fibroblast proliferation in the interlobular interstitium H.E. staining.

**Figure 5:**
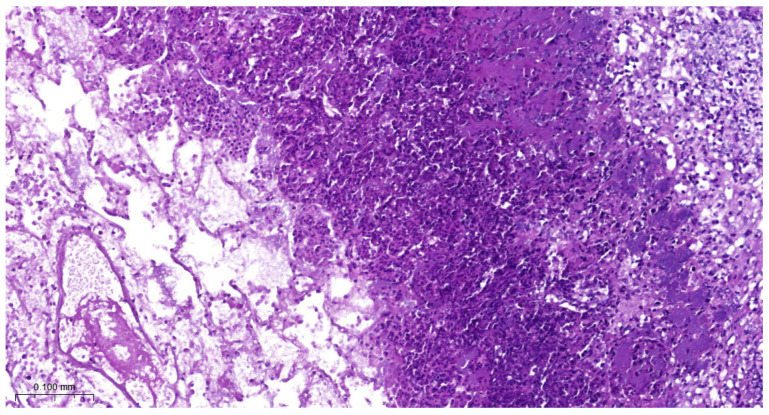
*A. pleuropneumoniae* serovar 16-infected lung on 6th day postinfection. Focal necrotic area in the lung surrounded by mononuclear inflammatory cells. Colonies of bacteria near to inflammatory cells. H.E. staining.

**Figure 6:**
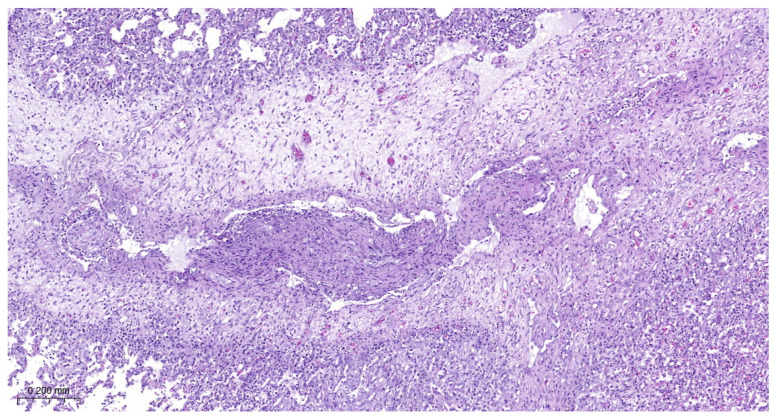
*A. pleuropneumoniae serovar* 16-infected lung on 6th day post infection. Fibroblast proliferation in the interlobular septum and organization of thrombus in a lymph vessel. H.E. staining.

Lung lesions were similar in all experimental groups. There were irregular necrotic-inflammatory foci in the lungs. In the necrotic area, fibrin thrombi were visible in the pulmonary capillaries. At the periphery of necrosis bacterial colonies and mononuclear cells (neutrophil granulocytes and macrophages) could be found. The outer layer of the foci was granulation tissue. The interlobular septa were wide, with fibroblasts proliferation. Dilated lymphatic vessels can be seen in these areas. There were also organized thrombi in the lymph vessels. There was bronchitis, edema, and alveolitis also seen in the lungs. Fibrinous pleurisy and organization of fibrine could also be observed (Figure 3, Figure 4, Figure 5 and Figure 6).

## 4. Discussion

There are remarkable differences in the virulence of *A. pleuropneumoniae* strains, and the presence and absence of virulence factors may explain them. Apx toxins are regarded to be the main virulence factors, and the ApxI, ApxII, and ApxIII toxins are produced in certain combinations, while the ApxIV toxin is produced by all *A. pleuropneumoniae* strains. The toxicity of these toxins is different: ApxI and ApxIII are strongly cytotoxic, while ApxII is moderately toxic [20], which can explain the differences in virulence. Quantification of other virulence factors, such as fimbriae, lipopolysaccharides, glycosphingolipids, outer membrane proteins, capsular polysaccharides, and transferrin-binding proteins as well as several enzymes, ability of biofilm formation, etc., is more difficult, and it is impossible to evaluate the interaction of the different virulence factors and judge the virulence of *A. pleuropneumoniae* in vitro [21,22,23,24,25,26,27,51]. Laboratory rodents have been used as infection models, but their pathogenesis does not represent that in pigs [52,53,54]. Thus, the most reliable method of evaluation of virulence is infection in pigs. Pigs were infected intranasally following the model of other authors which is close to the natural form of the infection [29,30,31]. The number of bacteria used by them in the infection were comparable to our doses.

*A. pleuropneumoniae* A-85/14 was described as type strain of serovar 16. Genes for the production (apxIA) and secretion (apxIB) of ApxI and the gene for the expression of ApxII and the largest size apxIV gene were detected in it, based on which a moderate-to-high virulence could be expected [44,45,46]. Highly virulent *A. pleuropneumoniae* strains frequently produce both ApxI and ApxIII toxins [20]. Our data show that *A. pleuropneumoniae* A-85/14 strain, a type strain of serovar 16, was capable of causing typical lesions in 12-week-old pigs when infected intranasally. Generally, no significant differences could be seen between the data of the three groups, and even 10^6^ cfu of *A. pleuropneumoniae* A-85/14 strain could induce clinical signs and typical lesions. However, the data show that the higher number of bacteria in the challenge solution cause more severe lesions and decreased body mass gain than the lower ones. Based on the presented data, the type strain of serovar 16 of *A. pleuropneumoniae* is considered to be a typical pathogenic strain of *A. pleuropneumoniae*.

## 5. Conclusions

Strain A-85/14, a type strain of *A. pleuropneumoniae* serovar 16, caused typical clinical signs and postmortem lesions of porcine pleuropneumonia; thus, this strain is considered to be a typical pathogenic strain of the species. The results prove that the challenge model presented in this paper is suitable for the assessment of vaccine efficacy against *A. pleuropneumoniae* serovar 16 in vaccine-challenge trials.

## Figures and Tables

**Table 1:** Scoring of clinical signs.

	Scores		
Clinical Signs	0	1	2
Weakness	No	Moderate	Intensive
Dyspnea	No	Moderate	Intensive
Respiratory rattle	No	Moderate	Intensive
Cough	No	Yes	-
Vomit	No	Yes	-
Anorexia	No	Yes	-

**Table 2:** Cumulative clinical scores, length of clinical signs, number of febrile days.

Groups	Diseased Animals	Average Clinical Score	Febrile Animals	Average Length of Clinical Signs	Average Number of Febrile Days
Group 1 (10^8^ cfu) *	10/10	12.60 ± 10.16 ^a^	5/10	4.20 ± 1.55 ^a^	0.90 ± 1.29 ^a^
Group 2 (10^7^ cfu)	9/10	8.70 ± 6.88 ^a,c^	4/10	4.00 ± 2.05 ^a^	0.50 ± 0.71 ^a^
Group 3 (10^6^ cfu)	7/9	3.89 ± 6.51 ^b,c^	2/9	1.89 ± 1.83 ^b^	0.33 ± 0.71 ^a^

* The different superscripts in the same column show significant difference (*p* < 0.05).

**Table 3:** Average body mass and mass gain.

Group	Body Mass (Day −1, kg)	Body Mass (Day 6, kg)	Mass Gain (kg)
Group 1 (10^8^ cfu) *	23.20 ± 2.54 ^a^	25.55 ± 3.58 ^a^	2.35 ± 1.92 ^a^
Group 2 (10^7^ cfu)	23.55 ± 1.83 ^a^	27.30 ± 2.87 ^a,c^	3.75 ± 2.69 ^a^
Group 3 (10^6^ cfu)	24.83 ± 2.44 ^a^	29.11 ± 2.93 ^b,c^	4.28 ± 2.20 ^a^

* The different superscripts in the same column show significant difference (*p* < 0.05).

**Table 4:** Postmortem lesions, postmortem scores, and re-isolation of the agent.

Group	Lung Lesions	Postmortem Score	Re-isolation of *A. pleuropneumoniae*
Group 1 (10^8^ cfu)	9/10	5.60 ± 6.08	8/9
Group 2 (10^7^ cfu)	8/10	4.50 ± 4.50	7/8
Group 3 (10^6^ cfu)	8/9	5.78 ± 6.85	7/8

## Data Availability

The data are contained within this paper and its Supplementary Table S1.

## Supplementary Materials

The following supporting information can be downloaded at: https://www.mdpi.com/article/10.3390/vetsci11020062/s1, Table S1: Row data of the examination of the virulence of *Actinobacillus pleuropneumoniae* serovar 16 in pigs.
